# Yield of testing and treatment for tuberculosis among foreign-born persons during contact investigations in the United States: A semi-systematic review

**DOI:** 10.1371/journal.pone.0200485

**Published:** 2018-07-19

**Authors:** Andrea Parriott, Mohsen Malekinejad, Amanda P. Miller, Hacsi Horvath, Suzanne M. Marks, James G. Kahn

**Affiliations:** 1 Philip R. Lee Institute for Health Policy Studies, University of California, San Francisco; San Francisco, California, United States of America; 2 Department of Epidemiology and Biostatistics, University of California, San Francisco; San Francisco, California, United States of America; 3 Division of Tuberculosis Elimination, Centers for Disease Control and Prevention, Atlanta, Georgia, United States of America; University of Sassari, ITALY

## Abstract

**Background:**

Contact investigation is an important strategy for maintaining control of tuberculosis (TB) in the United States. However, testing and treatment outcomes specifically to foreign-born populations are poorly understood. We reviewed literature on testing and LTBI identified during contact investigations in foreign-born populations living in the US.

**Methods:**

We conducted a comprehensive search of peer-reviewed and grey literature using Cochrane systematic review methods. We included studies with adult and adolescent populations that were at least 50% foreign-born. Pooled proportions and 95% confidence intervals (CIs) were calculated via inverse-variance weighted meta-analysis, and cumulative proportions were calculated as products of adjacent step proportions.

**Results:**

We identified 22 studies published between 1997 and 2014 that included at least 50% foreign-born participants. From studies of predominantly (>90%) foreign-born populations, almost all identified contacts were recruited and had valid test results, and 54.8% (95% CI 45.1–62.5%) of contacts with valid test results tested positive. From studies of majority (50% to 90%) foreign-born populations, 78.4% (95% CI 78.0–78.9%) of identified contacts were recruited, 92.0% (95% CI 91.6–92.3%) of recruited contacts had valid test results, and 38.5% (95% CI 31.9%-44.2%) of persons with valid results tested positive. These proportions varied by test type in studies of predominantly foreign-born populations. For every 1000 contacts identified in predominantly foreign-born populations, we estimate that 535 (95% CI 438 to 625) will test positive, and 354 (95% CI 244 to 453) will complete LTBI treatment. For every 1000 contacts identified in majority foreign-born populations, these estimates are 276 (95% CI 230 to 318), and 134 (95% CI 44 to 264), respectively.

**Conclusions:**

Contact investigation is a high yield activity for identifying and treating foreign-born persons with LTBI, but must be complemented by other tuberculosis control activities in order to achieve continued progress toward TB elimination.

## Introduction

In the United States, the majority of the burden of *Mycobacterium tuberculosis* infections occur in persons who were born in other countries. Tuberculosis (TB) infections can be divided into two types, latent TB infection (LTBI), an asymptomatic, non-communicable state, and TB disease (also referred to as active TB), which is symptomatic and contagious. TB disease is usually confined to the lungs, but disseminated and extrapulmonary disease are possible. Foreign-born persons accounted for approximately two thirds of the 9,557 cases of TB disease reported in the United States in 2015 [1]. From the 2012 National Health and Nutrition Examination Survey, 20.5% of the foreign-born participants over age 5 were tuberculin skin test (TST) positive [2]. A large number of TB cases occur among undocumented immigrants and persons in temporary visa classes [3, 4]. These undocumented and temporary foreign-born residents may be poorly integrated into the US medical care system, making them hard-to-reach for targeted testing and treatment for TB disease and LTBI.

There are currently two types of tests for LTBI. The first is the TST, where purified protein derivative (PPD) is injected into the forearm, and the injection site is examined 48 to 72 hours later for signs of an immune reaction. Because infection with other mycobacteria can cause an immune response to the antigens in PPD, TSTs may cause false positive results in persons exposed to nontuberculous mycobacteria or who received the Bacillus Calmette-Guerin (BCG) vaccine, a live virus vaccine of variable effectiveness among adults that is derived from an attenuated strain of *M*. *bovis*. BCG vaccine is not recommended for use in the United States because of low TB prevalence and false positive TST reactions among persons who received the vaccine. BCG vaccination is routine in many high TB burden countries, hampering the specificity of the TST in persons from these countries. The second type of test is the interferon gamma release assay (IGRA), which is a laboratory test performed on a blood sample. Second generation IGRA tests use antigens specific to *M*. *tuberculosis* and have superior specificity among BCG vaccinated persons, and by extension, persons born in high-incidence countries [5]. Neither test can distinguish between LTBI and TB disease; a diagnosis of LTBI requires a follow-up evaluation which may include symptom evaluation, chest radiographs, and sputum smears and cultures, to rule out TB disease.

Contact investigation is one of the major strategies used to maintain control of TB in the United States in which persons with contact to persons with infectious TB (i.e., contacts) are identified, recruited, tested, and treated for TB disease and LTBI [6]. While the primary goal of contact investigation is to identify additional cases of TB disease and to prevent TB disease incidence among contacts due to transmission from the index case, it also identifies persons who already have TB disease or have LTBI acquired from other index cases. This review seeks to examine the effectiveness of the continuum of care for TB contact investigations in recruiting, testing for TB infection using TST or IGRA, diagnosing LTBI and active TB among persons testing positive, and treating foreign-born adults and adolescents living in the United States.

## Materials and methods

We refer to this study as a “semi-systematic” review because it is an additional analysis of data derived from our systematic review of studies published between 1986 and 2014 examining the yield of community-based TB targeted testing and treatment programs in foreign born populations in the United States [7]. We used Cochrane Collaboration methods in all aspects of that review [8]. Studies were located using keywords related to tuberculosis, non-US birth, and occurring in the United States. See supplemental file (**S1 File)** for a complete description of the systematic review’s methods. In addition to reports of community-based TB targeted testing and treatment programs, search strategies captured studies relevant to TB contact investigation in the US. The current analysis includes all eligible reports of TB contact investigation identified through our systematic review, and methods used for screening, data extraction and analysis were identical to those used in the systematic review. The original search was performed on March 30, 2015. We updated the database search using the same databases and keywords on April 9, 2018.

### Eligibility criteria

In the current analysis, we include studies reporting the results of testing contacts of confirmed infectious TB cases. Testing for TB infection could be done by either TST or IGRA. Studies were eligible for inclusion if populations were adult or adolescent contacts of TB cases. Eligible studies reported including at least 50% foreign-born participants or provided sufficient data to suggest that such proportions were likely. In studies with less than 50% foreign-born participants overall, we included data from reported subgroups of at least 50% foreign-born participants. Data were stratified into two analysis groups: studies of contacts that were 50%-90% foreign-born (“majority foreign-born”; MFB), and studies of contacts that were more than 90% foreign-born (“predominantly foreign born”; PFB). Because the epidemiology of TB disease and LTBI differs in children and adults, we excluded studies conducted entirely in pediatric populations, but included studies with mixed adult and pediatric populations if children were a minority of the overall study population. Outputs of interest were eight sequential steps in the TB testing and treatment continuum of care 1) identification as a contact; 2) recruitment into the investigation (the contact was located and cooperated with the investigation); 3) obtaining a valid test result (reading of a TST, or laboratory processing and evaluation of an IGRA specimen); 4) testing positive for TB infection; 5) being diagnosed with active TB or LTBI during follow-up of positive test results (this does not include persons diagnosed with active TB who tested negative or were not tested for LTBI); 6) being offered LTBI treatment; 7) starting LTBI treatment; and 8) completing LTBI treatment. At a minimum, eligible studies reported numbers of contacts with valid test results and numbers of contacts that tested positive for TB infection.

### Data extraction and management

Two authors independently extracted data into a standardized, pre-piloted data extraction spreadsheet. Data extracted included the information included in Tables 1–3, the number of persons remaining at each step in the care cascade, and basic information about the index case **(S2 File)**. One author performed a first extraction of all data, and a second author checked the first author’s work and did a second extraction of all output variables while blinded to the first author’s extraction.

**Table 1:** Characteristics of TB contact investigations among foreign-born populations in the United States: Predominantly (>90%) foreign-born study populations.

Study	Setting (density)	Types of contacts	Period	Test type used	Contact countries or regions of origin (non-USA)	Index case countries or regions of origin	Number valid results
Albrecht 2004	California; Washington (rural)	Social	2003	TST	Mexico	Mexico	56
Brisette 2011	Harris County, Texas (urban)	Workplace, school, residence	2010–2011	IGRA or TST	Not reported	African countries	61
Dewan 2006	San Francisco, California (urban)	Workplace, school, residence	2004	TST	Mexico and Central American countries	Not reported, but not United States	43
Driver 2003	New York City, New York (urban)	Workplace, school	1995–2000	TST	Numerous	Numerous	1091
Gulati 2005	Not reported (not reported)	Workplace	2004	TST	Colombia, Ecuador, El Salvador, Guatemala, Honduras, India, Mexico, Peru, Poland	El Salvador	36
Ho 2010	San Francisco, California (urban)	Residence	2006–2007	TST	Mexico	Mexico	29
Kambali 2014	Texas (rural)	Workplace	2011	IGRA	Burma, Ethiopia, Haiti, Mexico, Somalia, Sudan, United States	Iraq	42
Kim 2002	Sussex County, Delaware (not reported)	Workplace, school, residence, social	Not reported	TST	Not reported	Guatemala, Mexico	82
Lowther 2011	Minnesota (rural)	Workplace, school, residence, social	2008	TST	El Salvador, Guatemala, Honduras, Mexico	Guatemala; United States	150
Person 2010	Wake County, North Carolina (urban)	Workplace	2005 or later	IGRA or TST	Not reported	Not reported	70
Smithee 2011	Oklahoma (not reported)	Workplace	2010	TST	Mexico (most)	Not reported	104
Wang 2010	Franklin County, Ohio (urban)	Workplace, school, residence, social contacts	Not reported, index cases diagnosed in 2006	IGRA or TST	Not reported	West Africa; Kenya	20

**Table 2:** Characteristics of TB contact investigations among foreign-born populations in the United States: Majority (50%-90%) foreign-born study populations.

Study	Setting (density)	Types of contacts	Period	Test type used	Contact countries or regions of origin (non-USA)	Index case countries or regions of origin	Number valid results
Miramontes 2010	Tennessee (not reported)	Workplace, residence, social contacts	2007–2009	TST	Guatemala	Guatemala	222
Rogers 2011	Greensboro, North Carolina (urban)	Workplace, school, residence, social contacts	2010	TST	Liberia	Not reported	89
Schack 2005	Colorado (rural)	Workplace, residence, social contacts	2004	TST	Not reported	Uganda	321
Trieu 2013	New York City, New York (urban)	Workplace, residence	2010–2011	IGRA	Not reported	Burma, Tibet	50
Yu 2011	Washington; Hawaii (both urban)	Residence	Not reported	IGRA or TST	Micronesia	Not reported	18

**Table 3:** Characteristics of TB contact investigations among foreign-born populations in the United States: Both majority and predominantly foreign-born study populations.

Study	Setting (density)	Types of contacts	Period	Test type used	Contact countries or regions of origin (non-USA)	Index case countries or regions of origin	Number valid results
Anger 2012	New York City, New York (urban)	Not reported	1997–2003	TST	Not reported	Many countries	25,164
Golub 2006	Maryland and other states in the region (not reported)	Workplace, school, residence, social contacts	Not reported; index cases diagnosed 2000–2001	TST	Many countries	Many countries	136
Grinsdale 2011	San Francisco, California (urban)	Workplace, school, residence, social contacts	Not reported; index cases diagnosed 2005–2007	IGRA or TST	Not reported	Many countries	1291
Marks, 2000	California, Georgia, Illinois, New Jersey, New York, Tennessee, Texas, Washington (all urban)	Not reported	1996–1997	TST	Not reported	Not reported	721
Ridzon, 1997	Southern California (not reported)	Workplace, school	1993–1994	TST	Vietnam	Mexico	1,109

### Statistical analysis and data synthesis

All data analyses were performed using Stata versions 12 and 14. Proportions of persons proceeding from one step in the testing and treatment cascade to subsequent steps were calculated for individual studies. Pooled summary proportions with 95% confidence intervals (CI) were estimated via inverse-variance weighted random effects meta-analysis. The Freeman-Tukey double arcsine transformation was used to normalize individual study proportions and ensure admissibility of proportion values of zero and one. Because TST and IGRA have different sensitivities and specificities, particularly in persons who have received the BCG vaccine, we calculated the following proportions stratified by test-type: positive tests of valid test results, diagnoses of TB disease of positive tests, and LTBI diagnoses of positive tests.

We also calculated pooled cumulative proportions for each step on the cascade, with the exception of active tuberculosis diagnoses, which we considered to be a “dead end” state because of very limited reporting of treatment data for those with active tuberculosis. Cumulative proportions were products of adjacent-step pooled proportions. Detailed methods for calculation of cumulative proportions have been described elsewhere [7].

### Risk of bias

We were unable to identify any appropriate tools for the assessment of the risk of bias or methodological quality in the individual studies. Existing standard instruments for assessing bias in intervention efficacy, prevalence, and other epidemiologic studies are not applicable to studies reporting the yield of screening programs [9, 10].

## Results

### Identified studies

**Fig 1:**
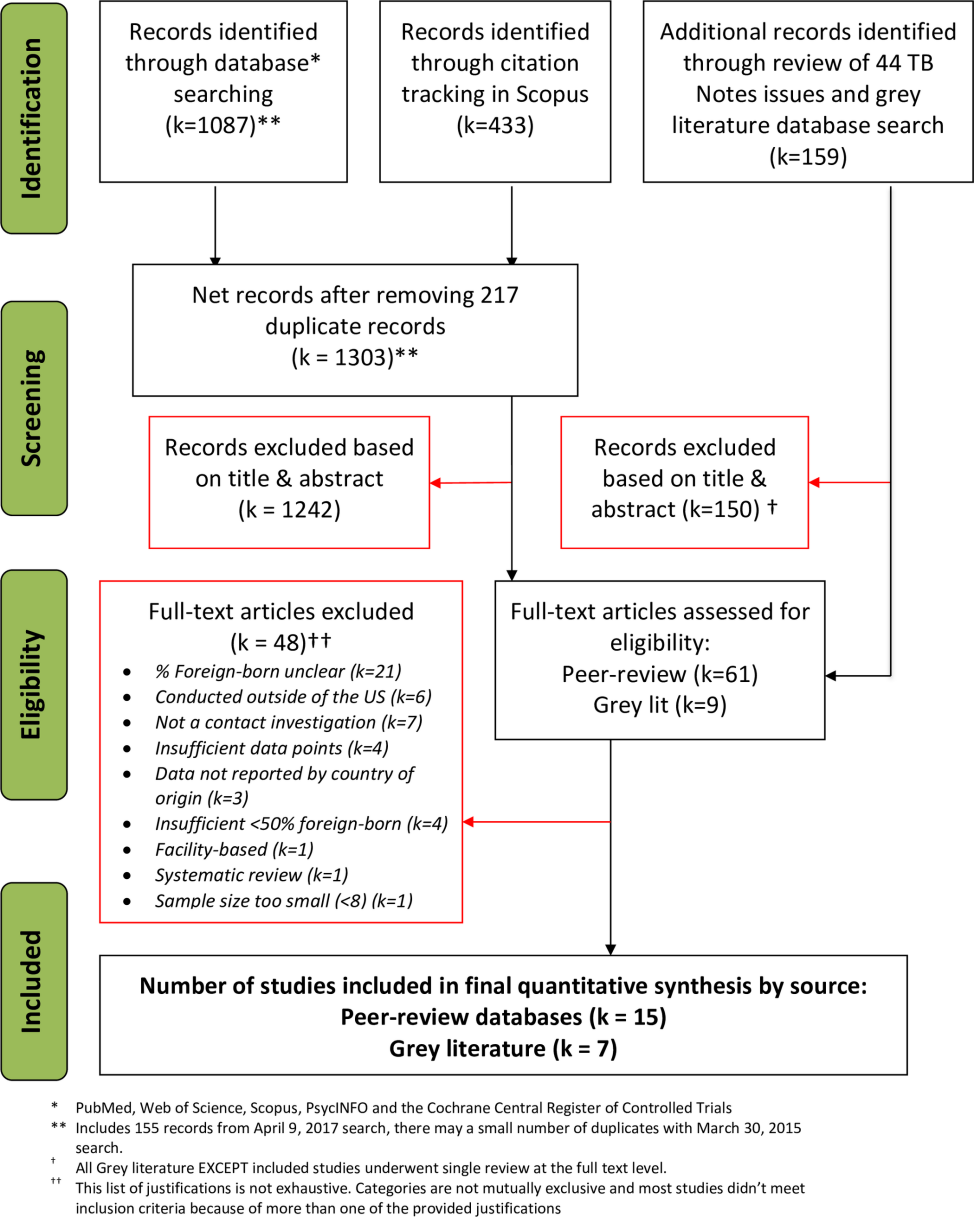
Identification and screening of citations: Semi-systematic review of contact investigation TB testing and treatment programs among foreign-born populations in the US.

The original search in March of 2015 yielded a total of 1,365 peer-reviewed citations and 159 grey literature reports (**Fig 1**), we identified 22 publications and conference abstracts that met our eligibility criteria. The search update in April of 2018 yielded 155 peer-reviewed citations, six of which were reviewed at the full text level. None of these were deemed eligible for inclusion. Because some article databases only allow date restriction by year, some studies published in early 2015 may have appeared in both searches. Descriptions of the included studies can be found in **Tables 1–3**. Studies described contact investigations in 16 different U.S. states (one did not identify the state), were published or presented between 1986 and 2014, and describe investigations conducted between 1982 and 2011. Foreign-born populations of contacts were diverse in national origin. Five [11–15] were aggregate reports of many contact investigations.

Identified contacts fell into three categories: household, workplace/school, and social contacts. The majority (13) of the studies were conducted at multiple sites and included contacts of more than one nature (e.g. colleagues, peers, familial) with variation in the hours of exposure with the index case. Nine studies were conducted among a single type of contact: six were workplace or school-based [16–21], two were residence-based [22, 23], and one included only social contacts [24]. Seven studies included contacts from all three categories [13–15, 18, 25]. All but one study reported the type of contacts [11].

### Author contacts

We did not identify any additional studies via author contact, however, one author provided additional unpublished data [26].

### Meta-analysis results

Among PFB populations in studies reporting numbers of persons identified and recruited, 98.8% (95% CI 94.7 to 100.0%) of identified contacts were recruited and 98.9% (95% CI 96.5 to 100.0%) of those recruited had valid test results. Proportions retained in these two steps were lower for studies in MFB populations; for the two studies reporting numbers identified and numbers recruited 78.4% (95% CI 78.0% to78.9%) of those identified were recruited and 92.0% (95% CI 91.6 to 92.3%) of those recruited had valid test results. Proportion testing positive of those with valid results was 54.8% (95% CI 45.3 to 64.1%) for PFB populations and 38.0% (95% CI 31.9 to 44.8%) for MFB populations. The vast majority of persons who tested positive were diagnosed with LTBI in both PFB and MFB populations Among studies reporting the number of active TB cases identified via LTBI testing, 5.5 percent (95% CI 1.2 to 11.7) of persons in PFB populations testing positive and 3.6% (95% CI 1.6 to 6.0%) of persons in MFB populations testing positive were diagnosed with TB disease. Treatment outcomes for persons with TB disease were rarely reported. Heterogeneity varied widely among the various proportions in the meta-analysis, but was generally high.

**Fig 2:**
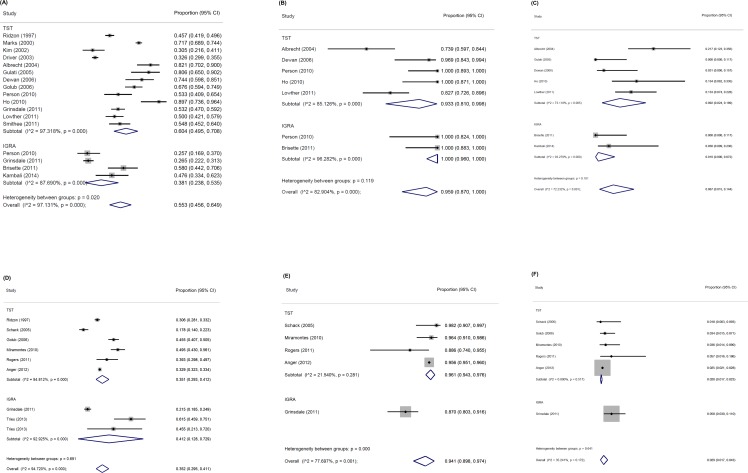
Forest plots for selected cascade proportions by test type and percent foreign-born.

**Fig 2** shows forest plots for the proportion testing positive of those with valid results and the proportion diagnosed with LTBI and TB disease of those testing positive, stratified by test type. For PFB populations, proportion testing positive was lower with IGRA than with TST (38.1% vs. 60.4%, P for difference 0.02), but for MFB populations, the point estimate of proportion testing positive by test type was similar (35.1% vs. 41.2%), but the confidence interval for IGRA testing was very wide (12.8% to 72.9%), meaning that substantial differences in proportion may be undetectable. The proportion ultimately diagnosed with TB disease among those testing positive was lower among those tested with IGRA (1.0% vs. 9.2% for TST) for PFB populations and higher among those tested with IGRA (5.8% vs. 2.0% for TST) for MFB populations, but neither difference was significant at α = 0.05. For PFB populations, the proportion diagnosed with LTBI of those with positive tests appeared similar across test types (although the confidence interval for those tested with IGRA was wide). Among MFB studies, the single study that used IGRA for testing and also reported the number diagnosed with LTBI found a lower proportion of LTBI diagnoses than the pooled proportion for MFB studies using TST.

**Fig 3:**
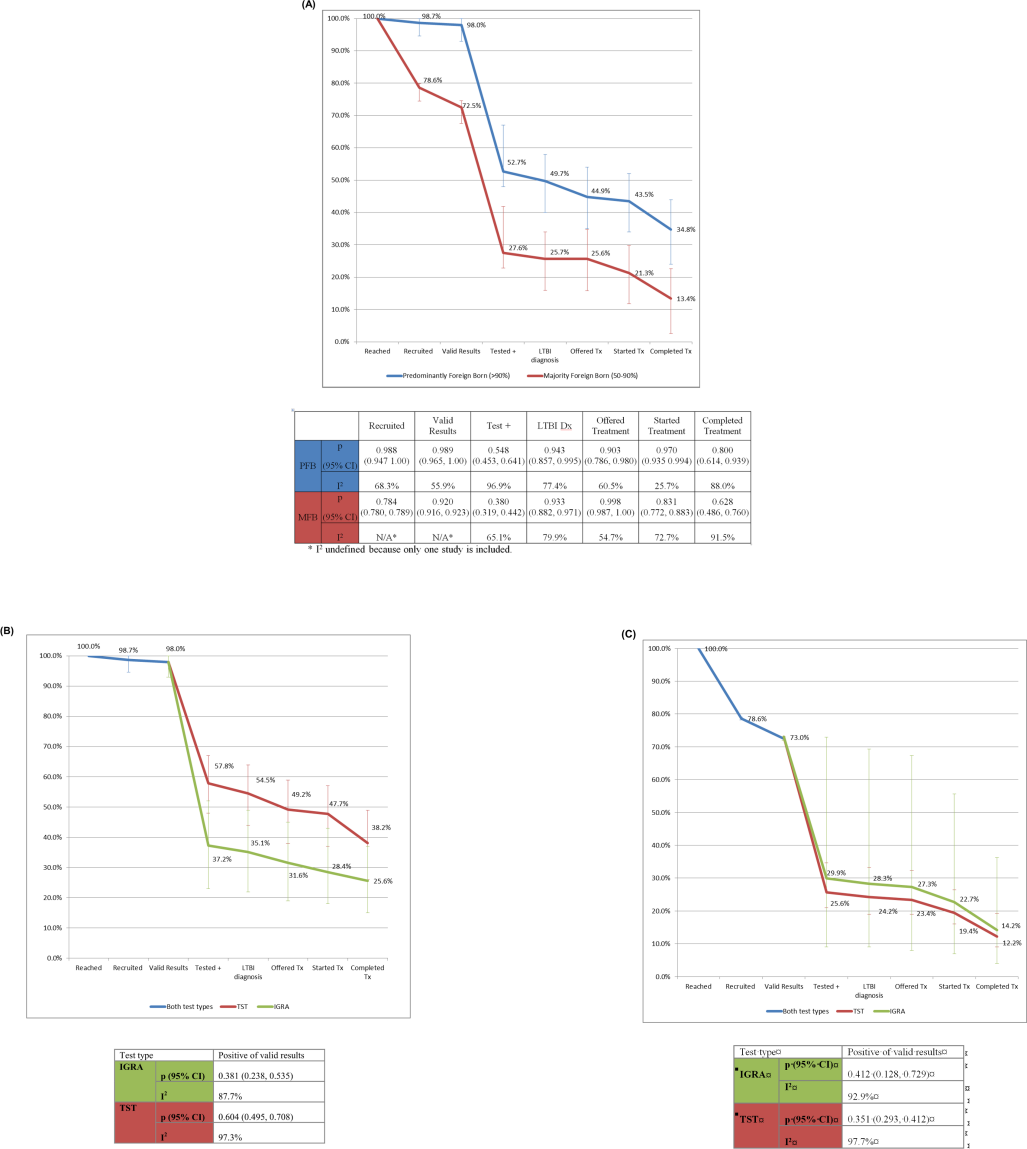
Cumulative proportion of participants retained in testing and treatment cascade in TB contact investigations in the United States, by percent foreign born and test type.

Adjacent step and cumulative proportions of those reaching each step in the testing and treatment cascade from identified contacts are shown in **Fig 3**. For every 1,000 contacts identified in PFB populations, we estimate that 535 (95% CI 437 to 625) will test positive, 505 (95% CI 405 to 596) will be diagnosed with LTBI, and 354 (95% CI 244 to 453) will complete LTBI treatment. For every 1,000 contacts in MFB populations we expect that 276 (95% CI 230 to 318), 213 (95% CI 173 to 250), and 134 (95% CI 44 to 264) will test positive, be diagnosed with LTBI, and complete LTBI treatment, respectively. The lower yield among MFB populations is due to a combination of lower recruitment rates, lower probability of testing positive, and lower retention in treatment among those diagnosed with LTBI and offered treatment. Among PFB, 70.1% (95% CI 52.1 to 84.2%) of those diagnosed with LTBI will complete LTBI treatment. Among MFB groups, 82.1% (95% CI 76.3 to 87.3%) of those testing positive will start LTBI treatment, but only 52.1% (95% CI 38.1 to 66.0%) of those diagnosed with LTBI will complete treatment. Looking only at use of TST among PFB populations, out of every 1,000 identified we estimate that the number testing positive, diagnosed with LTBI, and completing LTBI treatment will be 590 (95% CI 477 to 690), 557 (95% CI 439 to 657), and 390 (95% CI 266 to 489), while among those tested with IGRA, these numbers will be 371 (95% CI 233 to 517), 350 (95% CI 218 to 489), and 255 (95% CI 140 to 359), respectively. Because the proportion testing positive was similar for TST and IGRA among MFB populations, proportions were similar for all cumulative cascade proportions.

## Discussion

Our findings suggest that contact investigations conducted among mostly foreign-born populations yield a high proportion of detected infections and treatment completions per contact identified. This is particularly true for PFB populations, where patient cooperation rates with testing are in excess of 90% and the majority of identified contacts test positive.

In our previous work, we examined the yield of community-based testing programs targeting foreign-born persons who were not known to be contacts of infectious TB cases [7]. In that work, we found that, for every 100 persons targeted in PFB groups, 40 had valid test results, 16 tested positive, and 5 completed LTBI treatment. For MFB groups, these numbers were 80, 26, and 5, respectively. In this review, we found higher participation among those approached for testing, better retention through collection of valid test results, higher prevalence of positive tests, and higher proportions offered LTBI treatment. The higher proportion recruited and completing treatment may be because recent contacts of infectious cases are more motivated to cooperate with testing, because of more intensive efforts from contact investigation program staff, or both. Neither TST nor IGRA is able to distinguish between recent and remote infection, so for contacts diagnosed with LTBI, it is impossible to know whether they were actually infected by the contact investigation index case. Therefore, it is not clear whether TB infection prevalence is higher in this study because of transmission from the index case, or because contact investigations are better at targeting persons who are already at high risk of infection. It is also not clear why persons in contact investigations seem to be more likely to be offered treatment, but it may be due to the involvement of health department TB clinics, where treatment can be offered and followed up by knowledgeable staff.

There are a number of limitations of this study. Because of the lack of well accepted and widely used bias assessment tools for the type of studies in this review, we did not conduct a formal bias assessment. However, we can speculate on the types of biases that may have affected our results. Because contact investigations are conducted as public health operations rather than research, and public health practitioners are often too busy to write journal articles, there is publication bias in articles of contact investigation. While a recent CDC publication on routinely collected contact investigation indicators over a decade includes the care continuum steps included in this analysis, the indicators were not divided by US born and foreign-born contacts, so we have no way of assessing how published or presented results of investigations of foreign-born persons differ from those whose findings are never disseminated as published articles. Our estimates of the proportion of identified contacts diagnosed with LTBI (50.5% for PFB, 25.7% for MFB) are higher than the 20.5% diagnosed with LTBI among all 722,403 contacts evaluated and reported by 44 states and Puerto Rico to the CDC between 2003 and 2012 [27]. The differences are likely due to lower LTBI prevalence among US-born contacts, or to reporting or other bias in our estimates. The CDC report found a 46% rate of treatment completion among those diagnosed with LTBI that was similar to our 52% completion rate for MFB populations, but lower than the 70% seen for PFB. Misclassification is particularly problematic for data in the latter two steps in the cascade, number starting and completing treatment. Because directly observed therapy is generally recommended only for persons at high risk of default, most programs used self-administered therapy for LTBI treatment, and may have relied on self-report of initiation and completion of treatment, or used other proxies such as number of prescriptions filled. Also, persons may have been considered lost to follow-up if they left the country during treatment, even if they continued treatment abroad. Most studies did not provide adequate information about data collection practices to properly assess risk of misclassification. Interpretation of the proportion testing positive as the actual prevalence of tuberculosis infection should be done with caution, particularly for studies that used TST rather than IGRA, as sensitivity and specificity are imperfect and differ by test.

We found high heterogeneity in many of many of our proportions. The random effects model used to calculate the pooled proportions explicitly allows for heterogeneity of effect measure between studies. However, high heterogeneity indicates that the proportions proceeding from one cascade step to another are likely to vary widely between programs. Program planners, modelers, and others who may wish to use our results should consider the confidence intervals, which take heterogeneity into account, along with the pooled proportions.

Our intention was to examine testing for TB infection of adults and adolescents. However, some studies included children, and did not report results for children and adults separately. In those cases, the study groups included in the meta-analysis contain a small number of children. This is important because LTBI testing in children gives unreliable results, either because of false positivity due to infection with non-tuberculous mycobacteria or due to non-reactions because of developing immune systems in very young children. In addition, since TB infection identified through positive reactions to TST or IGRA, could indicate either TB disease or LTBI, we included both testing positive and LTBI diagnosis as steps in the cascade. We feel that this approach shows readers the difference between a positive test and LTBI diagnosis, both due to a small number of persons with TB disease and because some people do not complete follow-up evaluation to rule out TB disease.

Data collection for the investigations included in this review dated back to 1993, which leads to limited generalizability to future contact investigation outputs, given changes over time in both the prevalence of TB infection in the US and the technology available for TB testing and treatment. Of note, the majority of the studies we identified used TST, rather than IGRA, for testing. In part, this is because IGRA is a newer technology, with first generation tests first licensed in 2001, and more specific second generation tests first licensed in 2005. However, even among the thirteen studies reporting investigations known or suspected to be conducted after 2005, five wholly relied on TST rather than IGRA and four used a combination of TST and IGRA. If IGRA eventually becomes the standard test for investigation of foreign born contacts, the proportion of persons testing positive of those with valid results will likely be lower than estimated here, but persons testing positive will also be more likely to be truly infected, lowering the number needed to treat to prevent one case of active TB. Also, no studies were published in 2012 or later. This is important because the US Centers for Disease Control and Prevention issued guidelines in December of 2011 for use of a 12-dose, 3-month combined isoniazid and rifapentine treatment regimen (known as 3HP) provided by directly observed therapy, which has been documented to have greater completion rates (~80%) than longer (6 to 9 month) isoniazid treatment regimens [28]. As 3HP becomes the standard of care for LTBI treatment, this study documents historical treatment completion for comparison.

## Conclusions

Contact investigations appear to be an effective means of recruiting FB persons for targeted TB testing and treatment. However, while there are only a few thousand cases of active TB per year in the US, there are millions of persons with LTBI living in the United States, most of whom were infected abroad. Contact investigations alone clearly cannot be relied upon to reach the majority of at-risk foreign-born persons, and must be supplemented with other targeted testing activities, such as community-based targeted testing programs and pre-arrival testing and treatment of US-bound immigrants [7, 29], if sustained declines in tuberculosis incidence and prevalence are to be achieved.

## Supporting information

S1 File Database search strategies. (DOCX)

S2 File Data extraction sheet. (CSV)

S3 File Articles screened at the full-text level. (DOCX)

S4 File Proportion and 95% confidence interval for TB testing and treatment cascade. (DOCX)

S5 File Prisma checklist. (DOCX)
