# Supplementary material for: Yield of testing and treatment for tuberculosis among foreign-born persons during contact investigations in the United States: A semi-systematic review
Source: PLoS One. 2018 Jul 19;13(7):e0200485. doi: 10.1371/journal.pone.0200485 (PMC6053151; doi:10.1371/journal.pone.0200485)
Supplement: S3 File — (DOCX) [file pone.0200485.s003.docx]

**S3: Articles screened at the full text level**

**Systematic Review of contact investigations among foreign born individuals in the United States to find and treat active and latent tuberculosis infection (LTBI): UCSF**

**CAPE Project**

Contents

Included (k=22) 1

Excluded: % foreign born unclear (N=18) 3

Excluded: Conducted outside of the United States (k=5) 4

Excluded: Not a contact investigation (k=4) 5

Excluded: Insufficient data points (k=4) 5

Excluded: Data not reported by country of origin (N=3) 5

Excluded: Insufficient % foreign born (<50%) (k=3) 6

Excluded: Facility-based (k=1) 6

Excluded: Systematic review (k=1) 6

Excluded: Sample size too small (<8) (k=1) 6

## Included (k=22)

#### Peer Reviewed Literature (k=15)

Anger HA, Proops D, Harris TG, Li J, Kreiswirth BN, Shashkina E, et al. Active case finding and prevention of tuberculosis among a cohort of contacts exposed to infectious tuberculosis cases in New York City. Clinical Infectious Disease. 2012;54(9):1287-95.

Dewan PK, Banouvong H, Abernethy N, Hoynes T, Diaz L, Woldemariam M, et al. A tuberculosis outbreak in a private-home family child care center in San Francisco, 2002 to 2004. Pediatrics. 2006;117(3):863-9.

Driver C.R., Balcewicz-Sablinska M.K., Kim Z., Scholten J., Munsiff S.S. Contact investigations in congregate settings, New York City. International Journal of Tuberculosis and Lung Disease. 2003;7(12 Suppl 3):S432-8.

Golub J.E., Bur S., Cronin W.A., Gange S., Baruch N., Comstock GW, et al. Delayed tuberculosis diagnosis and tuberculosis transmission. International Journal of Tuberculosis and Lung Disease. 2006;10(1):24-30.

Grinsdale J.A., Ho C.S., Banouvong H., Kawamura L.M. Programmatic impact of using QuantiFERON(R)-TB Gold in routine contact investigation activities. International Journal of Tuberculosis and Lung Disease. 2011;15(12):1614-20.

Gulati M., Liss D.J., Sparer J.A., Slade M.D., Holt E.W., Rabinowitz P.M. Risk factors for tuberculin skin test positivity in an industrial workforce results of a contact investigation. Journal of Occupational and Environmental medicine / American College of Occupational and Environmental Medicine. 2005;47(11):1190-9.

Kambali S., Nantsupawat N., Lee M., Nugent K. A Workplace Tuberculosis Case Investigation in the Presence of Immigrant Contacts from High Prevalence Countries. Journal of Community Health. 2014.

Kim D.Y., Ridzon R., Giles B., Mireles T. Pseudo-outbreak of tuberculosis in poultry plant workers, Sussex County, Delaware. Journal of Occupational and Environmental Medicine. 2002;44(12):1169-72.

Lowther S.A., Miramontes R., Navara B., Sabuwala N., Brueshaber M., Solarz S., et al. Outbreak of tuberculosis among Guatemalan immigrants in rural Minnesota, 2008. Public Health Reports (Washington, DC : 1974). 2011;126(5):726-32.

Marks S.M., Taylor Z., Qualls N.L., Shrestha-Kuwahara R.J., Wilce M.A., Nguyen C.H. Outcomes of contact investigations of infectious tuberculosis patients. American Journal of Respiratory and Crit Care Medicine. 2000;162(6):2033-8.

Miramontes R., Lambert L., Haddad M.B., Boaz V., Hawkins S, Zylstra M, et al. Public Health Response to a Multidrug-Resistant Tuberculosis Outbreak Among Guatemalans in Tennessee, 2007. Southern Medical Journal. 2010;103(9):882-6.

Person A.K., Goswami N.D., Bissette D.J., Turner D.S., Baker A.V., Gadkowski L.B., et al. Pairing QuantiFERON gold in-tube with opt-out HIV testing in a tuberculosis contact investigation in the Southeastern United States. AIDS Patient Care and STDs. 2010;24(9):539-43.

Ridzon R., Kent J.H., Valway S., Weismuller P., Maxwell R, Elcock M, et al. Outbreak of drug-resistant tuberculosis with second-generation transmission in a high school California. Journal of Pediatrics. 1997;131(6):863-8.

Trieu L., Proops D.C., Ahuja S.D. Using QuantiFERON-TB gold in-tube for field-based tuberculosis contact investigations in congregate settings. Journal of Public Health Management and Practice. 2013;19(3):E11-E3.

Wang S.H., Hunt WG, Powell DA. Lessons learned from two school tuberculosis investigations. Journal of Immigrant and Minority Health / Center for Minority Public Health. 2010;12(6):853-8.

#### Grey literature: TB Notes (k=3)

Albrecht T. A Multi-Jurisdictional TB Outbreak Among Seasonal Agricultural Workers From Oaxaca, Mexico. TB Notes. 2004(2):4-6.

Ho C. TB Outreach Among Indigenous Mexican Immigrants in San Francisco. TB Notes. 2010(3):7-10.

Rogers B. Contact Investigation Among Liberian Refugees in Greensboro, North Carolina. TB Notes. 2011(2):8-9.

Schack G. Misdiagnosis in Rural Colorado TB Notes. 2005(2):6-8.

#### Conference Proceedings (k=4)

Brisette B. Challenges and Innovative Solutions in the Case Management of Immigrant Patients. National Tuberculosis Controllers Association Annual Meeting; Atlanta, Georgia. 2011.

Smithee L. Application of IGRA to a Contact Investigation Involving a Predominant Mexican Population in a Worksite 7th Annual Conference on Laboratory Aspects of Tuberculosis; Wednesday, June 15, 2011; Atlanta, Georgia. 2011.

Yu D. Cultural considerations in TB contact investigation. 2011 National TB Conference; June 15-17, 2011; Atlanta, Georgia. 2011.

## Excluded: % foreign born unclear (N=21)

Bates JH, Potts WE, Lewis M. Epidemiology of primary tuberculosis in an industrial school. The New England journal of medicine. 1965;272:714-7.

Behr MA, Hopewell PC, Paz EA, Kawamura LM, Schecter GF, Small PM. Predictive value of contact investigation for identifying recent transmission of Mycobacterium tuberculosis. American Journal of Respiratory and Critical Care Medicine. 1998;158(2):465-9.

Camden TL, Maruffo D, Santos N, Nava JJ, Alcantara C: Investigation of Tuberculosis in a High School - San Antonio, Texas, 2012. MMWR Morbidity and mortality weekly report 2015, **64**(31):856.

CDC. Interstate outbreak of drug-resistant tuberculosis involving children--California, MOntana, Nevada, Utah. MMWR. 1983;39(516-8).

CDC. Outbreak of multidrug-resistant tuberculosis-Texas, California, and Pennsylvania. MMWR. 1990;39:369-72.

Davidow AL, Mangura BT, Wolman MS, Bur S, Reves R, Thompson V, et al. Workplace contact investigations in the United States. International Journal of Tuberculosis and Lung Disease. 2003;7(12 SUPPL. 3):S446-S52.

Golub JE, Cronin WA, Obasanjo OO, Coggin W, Moore K, Pope DS, et al. Transmission of Mycobacterium tuberculosis through casual contact with an infectious case. Archives of Internal Medicine. 2001;161(18):2254-8.

Gross TP, Silverman PR, Bloch AB, Smith TY, Rogers GW. An outbreak of tuberculosis in rural Delaware. Am J Epidemiol. 1989;129(2):362-71.

Hoge CW, Fisher L, Donnell HD, Jr., Dodson DR, Tomlinson GV, Jr., Breiman RF, et al. Risk factors for transmission of Mycobacterium tuberculosis in a primary school outbreak: lack of racial difference in susceptibility to infection. Am J Epidemiol. 1994;139(5):520-30.

Kaupas V. Tuberculosis in a family day-care home. Report of an outbreak and recommendations for prevention. Journal of the American Medical Association. 1974;228(7):851-4.

Lobato MN, Royce SE, Mohle-Boetani JC. Yield of source-case and contact investigations in identifying previously undiagnosed childhood tuberculosis. International Journal of Tuberculosis and Lung Disease. 2003;7(12 SUPPL. 3):S391-S6.

Mangura BT, Napolitano EC, Passannate MR, McDonald RJ, Reichman LB. Mycobacterium tuberculosis miniepidemic in a church gospel choir. Chest. 1998;113(1):234-7.

Mitruka K, Oeltmann JE, Ijaz K, Haddad MB. Tuberculosis outbreak investigations in the United states, 2002-2008. Emerging Infectious Diseases. 2011;17(3):425-31.

Mosher CB, Derebery VJ, Young BJ, Adams RA. Unusually aggressive transmission of tuberculosis in a factory. Journal of Occupational Medicine. 1987;29(1):29-31.

Muñoz FM, Ong LT, Seavy D, Medina D, Correa A, Starke JR. Tuberculosis Among Adult Visitors of Children With Suspected Tuberculosis and Employees at a Children's Hospital. Infection Control & Hospital Epidemiology. 2002;23(10):568-72.

Phillips L, Carlile J, Smith D. Epidemiology of a tuberculosis outbreak in a rural Missouri high school. Pediatrics. 2004;113(6):e514-9.

Reves R, Blakey D, Snyder DE, Farer L. Transmission of multiple drug-resistant tuberculosis: report of a school and community outbreak American Journal of Epidemiology. 1981;113(4).

Rothfeldt LL, Patil N, Haselow DT, Williams SH, Wheeler JG, Mukasa LN: Notes from the Field: Cluster of Tuberculosis Cases Among Marshallese Persons Residing in Arkansas - 2014-2015. MMWR Morbidity and Mortality Weekly Report 2016, 65(33):882-883.

Weinberg MP, Cherry C, Lipnitz J, Nienstadt L, King-Todd A, Haddad MB, Russell M, Wong D, Davidson P, McFadden J et al: Tuberculosis Among Temporary Visa Holders Working in the Tourism Industry - United States, 2012-2014. MMWR Morbidity and Mortality Weekly Report 2016, 65(11):279-281.

## Excluded: Conducted outside of the United States (k=6)

Diel R, Loddenkemper R, Meywald-Walter K, Gottschalk R, Nienhaus A. Comparative performance of tuberculin skin test, Quanti FERON-TB-Gold in tube assay, and T-SpotTB test in contact investigations for tuberculosis. Chest. 2009;135(4):1010-8.

Grzybowski S, Barnett GD, Styblo K. Contacts of cases of active pulmonary tuberculosis. Bulletin of the International Union against Tuberculosis. 1975;50(1):90-106.

Higuchi K, Harada N, Mori T, Sekiya Y. Use of QuantiFERON®-TB Gold to investigate tuberculosis contacts in a high school. Respirology. 2007;12(1):88-92.

Hyge T. Epidemic of tuberculosis in a state school, with an observation period of about 3 years. Acta Tuberculosis Scand. 1947;21:1-57.

Wiseman CA, Mandalakas AM, Kirchner HL, Gie RP, Schaaf HS, Walters E, et al. Novel application of NIH case definitions in a paediatric tuberculosis contact investigation study. International Journal of Tuberculosis and Lung Disease. 2015;19(4):446-53.

Whatney WE, Gandhi NR, Lindestam Arlehamn CS, Nizam A, Wu H, Quezada MJ, Campbell A, Allana S, Kabongo MM, Khayumbi J et al: A High Throughput Whole Blood Assay for Analysis of Multiple Antigen-Specific T Cell Responses in Human Mycobacterium tuberculosis Infection. Journal of Immunology (Baltimore, Md : 1950) 2018.

## Excluded: Not a contact investigation (k=7)

Behr MA, Warren SA, Salamon H, Hopewell PC, Ponce De Leon A, Daley CL, et al. Transmission of Mycobacterium tuberculosis from patients smear-negative for acid-fast bacilli. Lancet. 1999;353(9151):444-9.

Cronin WA, Golub JE, Lathan MJ, Mukasa LN, Hooper N, Razeq JH, et al. Molecular epidemiology of tuberculosis in a low-to moderate-incidence state: Are contact investigations enough? Emerging Infectious Diseases. 2002;8(11):1271-9.

Giles N, Bhatia R: Cough and fever in an immigrant adolescent with abnormal chest X-ray. Respiratory Medicine Case Reports 2017, 20:19-21.

Haddad MB, Mitruka K, Oeltmann JE, Johns EB, Navin TR. Characteristics of tuberculosis cases that started outbreaks in the united states, 2002–2011. Emerging Infectious Diseases. 2015;21(3):508-10.

Klovdahl AS, Graviss EA, Yaganehdoost A, Ross MW, Wanger A, Adams GJ, et al. Networks and tuberculosis: an undetected community outbreak involving public places. Social Science and Medicine. 2001;52(5):681-94.

Soren K., Saiman L., Irigoyen M., Gomez-Duarte C., Levison MJ, McMahon DJ. Evaluation of household contacts of children with positive tuberculin skin tests. Pediatric Infectious Disease Journal. 1999;18(11):949-55.^[[1]](#footnote-1)^

Sullam P.M., Slutkin G., Hopewell P.C.. The benefits of evaluating close associates of child tuberculin reactors from a high prevalence group. American Journal of Public Health. 1986;76(9):1109-11.

## Excluded: Insufficient data points (k=4)

Bennett DE, Onorato IM, Ellis BA, Crawford JT, Schable B, Byers R, et al. DNA fingerprinting of Mycobacterium tuberculosis isolates from epidemiologically linked case pairs. Emerg Infect Dis. 2002;8(11):1224-9.

Li J, Marks SM, Driver CR, Diaz FA, Castro AF, 3rd, de Regner AF, et al. Human immunodeficiency virus counseling, testing, and referral of close contacts to patients with pulmonary tuberculosis: feasibility and costs. J Public Health Manag Pract. 2007;13(3):252-62.

Mitruka K, Blake H, Ricks P, Miramontes R, Bamrah S, Chee C, et al. A tuberculosis outbreak fueled by cross-border travel and illicit substances: Nevada and Arizona. Public health reports (Washington, DC : 1974). 2014;129(1):78-85.

Wadell R. Bilingual-Access Phone Cards: An Incentive to Completing LTBI Treatment Among Foreign-born Persons, Alabama, 2001 TB Notes. 2002(3):10-1.

## Excluded: Data not reported by country of origin (N=3)

Driver CR. Transmission of Mycobacterium tuberculosis Associated With Air Travel. JAMA: The Journal of the American Medical Association. 1994;272(13):1031.

Driver CR, Cordova IM, Munsiff SS. Targeting tuberculosis testing: The yield of source case investigations for young children with reactive tuberculin skin tests. Public Health Reports. 2002;117(4):366-72.

Linquist JA, Rosaia CM, Riemer B, Heckman K, Alvarez F. Tuberculosis exposure of patients and staff in an outpatient hemodialysis unit. American Journal of Infection Control. 2002;30(5):307-10.

## Excluded: Insufficient % foreign born (<50%) (k=4)

Braden CR, Valway SE, Onorato IM, Ussery XT, Grant SB, Dwyer D, et al. Infectiousness of a university student with laryngeal and cavitary tuberculosis. Clinical Infectious Diseases. 1995;21(3):565-70.

Curtis AB, Ridzon R, Vogel R, McDonough S, Hargreaves J, Ferry J, et al. Extensive transmission of Mycobacterium tuberculosis from a child. New England Journal of Medicine. 1999;341(20):1491-5.

Hirsch-Moverman Y, Cronin WA, Chen B, Moran JA, Munk E, Reichler MR: HIV counseling and testing in tuberculosis contact investigations in the United States and Canada. The International Journal of Tuberculosis and Lung Disease, 19(8):943-953.

Pevzner ES, Robison S, Donovan J, Allis D, Spitters C, Friedman R, et al. Tuberculosis transmission and use of methamphetamines in Snohomish County, WA, 1991-2006. Am J Public Health. 2010;100(12):2481-6.

## Excluded: Facility-based (k=1)

CDC. Nosocomial transmission of multidrug-resistant tuberculosis among HIV-infected persons--Florida and New York, 1988-1991. Morbidity and mortality weekly report. 1991;40(34):585-91.

## Excluded: Systematic review (k=1)

Shah NS, Yuen CM, Heo M, Tolman AW, Becerra MC. Yield of contact investigations in households of patients with drug-resistant tuberculosis: Systematic review and meta-analysis. Clinical Infectious Diseases. 2014;58(3):381-91.

## Excluded: Sample size too small (<8) (k=1)

Moonan PK, Quitugua T, Cox RA, Weis SE. Associate investigations: detection of tuberculosis infections in children resulting in discovery of undiagnosed tuberculosis in adults. The Journal of the American Osteopathic Association. 2002;102(7):397-400.

1. These references are source-case investigations, which differ from contact investigations in that the index case has LTBI rather than TB disease, and the goal is to find the source of the infection, rather than persons infected by the index case. [↑](#footnote-ref-1)
