# Supplementary material for: Yield of testing and treatment for tuberculosis among foreign-born persons during contact investigations in the United States: A semi-systematic review
Source: PLoS One. 2018 Jul 19;13(7):e0200485. doi: 10.1371/journal.pone.0200485 (PMC6053151; doi:10.1371/journal.pone.0200485)
Supplement: S4 File — (DOCX) [file pone.0200485.s004.docx]

**S4: Proportion and 95% confidence interval for TB testing and treatment cascade: contact investigation programs among foreign-born populations in the US.**

Proportion proceding from each step to each subsequent step, calculated directly. Cumulative proportions are products of the proportions in the diagonal row. (A) Predominantly (>90%) foreign-born populations. (B) Majority (50-90%) foreign-born populations.

|  |  |  |  |  |  | **LTBI** | | |
| --- | --- | --- | --- | --- | --- | --- | --- | --- |
|  | **Recruited** | **Valid**  **Results** | **Test (+)** | **Active TB** | **LTBI Dx** | **Offered**  **Treatment** | **Started**  **Treatment** | **Completed**  **Treatment** |
| **Identified** | **0.99** | **0.91** | **0.55** | **0.04** | **0.51** | **0.45** | **0.46** | **0.35** |
|  | **(0.95, 1.00)** | **(0.81, 0.98)** | **(0.48, 0.63)** | **(0.01, 0.09)** | **(0.40, 0.62)** | **(0.38, 0.53)** | **(0.38, 0.54)** | **(026, 0.45)** |
| **Recruited** |  | **0.99** | **0.52** | **0.01** | **0.47** | **0.35** | **0.49** | **0.40** |
|  |  | **(0.97, 1.00)** | **(0.40, 0.63)** | **(0.00, 0.05)** | **(0.31, 0.63)** | **(0.23, 0.48)** | **(0.29, 0.69)** | **(0.23, 0.59)** |
| **Valid**  **Results** |  |  | **0.54** | **0.03** | **0.53** | **0.46** | **0.53** | **0.38** |
|  |  |  | **(0.45, 0.62)** | **(0.00, 0.07)** | **(0.39, 0.66)** | **(0.35, 0.58)** | **(0.41, 0.65)** | **(0.30, 0.48)** |
| **Test ( +)** |  |  |  | **0.04** | **0.94** | **0.91** | **0.81** | **0.60** |
|  |  |  |  | **(0.00, 0.11)** | **(0.86, 1.0)** | **(0.79, 0.99)** | **(0.73, 0.88)** | **(0.50, 0.70)** |
| **LTBI Dx** |  |  |  |  |  | **.90** | **0.81** | **0.67** |
|  |  |  |  |  |  | **(0.79 0.98)** | **(0.71, 0.90)** | **(0.49, 0.82)** |
| **Offered  treatment** |  |  |  |  |  |  | **0.97** | **0.68** |
|  |  |  |  |  |  |  | **(0.93 , 0.99)** | **(0.55, 0.79)** |
| **Started  treatment** |  |  |  |  |  |  |  | **0.80** |
|  |  |  |  |  |  |  |  | **(0.61, 0.94)** |
| **Legend** | **1 Study** | | **2 Studies** | | **3-4 Studies** | | **> 4 Studies** | |

**B.**

|  |  |  |  |  |  | **LTBI** | | |
| --- | --- | --- | --- | --- | --- | --- | --- | --- |
|  | **Recruited** | **Valid**  **Results** | **Test (+)** | **Active TB** | **LTBI Dx** | **Offered  Treatment** | **Started  Treatment** | **Completed  Treatment** |
| **Identified** | **0.79** | **0.78** | **0.29** | **0.01** | **0.21** | **0.23** | **0.16** | **0.10** |
|  | **(0.78, 0.79)** | **(0.66, 0.87)** | **(0.22, 0.36)** | **(0.00, 0.01)** | **(0.18, 0.25)** | **(0.22, 0.23)** | **(0.13, 0.21)** | **(0.10, 0.11)** |
| **Recruited** |  | **.92** | **0.30** | **0.01** | **0.29** | **0.29** | **0.23** | **0.13** |
|  |  | **(0.92, 0.93)** | **(0.30, 0.31)** | **(0.01, 0.01)** | **(0.28, 0.29)** | **(0.28, 0.29)** | **(0.22, 0.23)** | **(0.13, 0.14)** |
| **Valid**  **Results** |  |  | **0.38** | **0.01** | **0.32** | **0.31** | **0.24** | **0.15** |
|  |  |  | **(0.32, 0.44)** | **(0.00, 0.02)** | **(0.24, 0.41)** | **(0.25, 0.38)** | **(0.17, 0.31)** | **(0.13, 0.17)** |
| **Test** |  |  |  | **0.04** | **0.93** | **0.96** | **0.76** | **0.48** |
| **(+)** |  |  |  | **(0.02, 0.06)** | **(0.88, 0.97)** | **(0.90, 1.00)** | **(0.75, 0.77)** | **(0.38, 0.58)** |
| **LTBI Dx** |  |  |  |  |  | **1.00** | **0.82** | **0.52** |
|  |  |  |  |  |  | **(0.99, 1.00)** | **(0.76, 0.87)** | **(0.38, 0.66)** |
| **Offered  treatment** |  |  |  |  |  |  | **0.83** | **0.53** |
|  |  |  |  |  |  |  | **(0.77, 0.88)** | **(0.38, 0.67)** |
| **Started  treatment** |  |  |  |  |  |  |  | **0.63** |
|  |  |  |  |  |  |  |  | **(0.49, 0.76)** |
| **Legend** | **1 Study** | | **2 Studies** | | **3-4 Studies** | | **> 4 Studies** | |
